# Acute administration of alprazolam, alcohol and their combination on cognitive performance and mood: A randomised, double-blind, placebo-controlled study

**DOI:** 10.1177/02698811231200878

**Published:** 2023-09-19

**Authors:** Blair Aitken, Amie C Hayley, Talitha C Ford, Lauren Geier, Brook A Shiferaw, Luke A Downey

**Affiliations:** 1 Centre for Mental Health and Brain Sciences, Swinburne University of Technology, Hawthorn, VIC, Australia; 2 Institute for Breathing and Sleep, Austin Health, Heidelberg, VIC, Australia; 3 Cognitive Neuroscience Unit, Deakin University, Geelong, VIC, Australia; 4 Forensic Science SA, Adelaide, SA, Australia; 5 Seeing Machines Ltd., Fyshwick, ACT, Australia

**Keywords:** Alprazolam, alcohol, benzodiazepines, cognitive performance, randomised controlled trial

## Abstract

**Background:**

Recreational co-consumption of benzodiazepines and alcohol is a common practise; yet, the cognitive effects of this combination remain poorly understood. This study aimed to investigate the acute cognitive effects of combining a 1 mg dose of alprazolam with a moderate dose of alcohol (target 0.04% blood alcohol concentration (BAC)) in a non-clinical population.

**Methods:**

In this randomised, double-blind, placebo-controlled, crossover trial, participants completed computerised cognitive assessments and the brief biphasic alcohol effects scale (B-BAES) after consuming 1 mg of alprazolam, both with and without a moderate dose of alcohol (target 0.04% BAC).

**Results:**

Among 20 healthy participants (mean age = 28.6, SD ± 4.0 years, 60% female), we found that a peak BAC of 0.03% had no significant impact on cognitive performance. Both the individual use of alprazolam and its combination with alcohol resulted in impaired reaction time, digit vigilance, and verbal, spatial and numeric working memory tasks, although an additive effect when alcohol and alprazolam were consumed together was not evident. The most pronounced cognitive effects occurred at 100 min after dosing, coinciding with increased alprazolam concentrations. Sedative effects were heightened with alcohol, alprazolam and their combination while no stimulative effects were reported.

**Conclusions:**

Our findings highlight the significant implications of a therapeutic dose of alprazolam on impairing cognitive performance. This is particularly relevant considering the frequency of non-medical alprazolam use. Future studies should explore different dosages, administration timings and long-term effects to inform the development of public health policies and guidelines regarding the combined use of alcohol and benzodiazepines.

## Introduction

Central nervous system depressants, including alcohol (Garrisson et al., 2021), recreational drugs (e.g. cannabis; McCartney et al., 2021), and both prescription (e.g. benzodiazepines; Aitken et al., 2021) and over-the-counter medications (e.g. antihistamines; Saxena et al., 2020), negatively affect various aspects of cognitive function. Among these substances, alcohol has been extensively examined due to its widespread use and profound impact on public health, contributing to the global burden of disease, injury and economic costs (World Health Organization, 2018). Evidence of clinically relevant deficits in divided attention, psychomotor function, visual perception and vigilance often emerges at blood alcohol concentrations (BACs) below 0.05%. Conversely, executive function and reaction time are less susceptible, with deficits occurring at BACs of 0.08% and higher (Garrisson et al., 2021). This research indicates that several aspects of cognitive function become impaired, even at relatively moderate doses of alcohol. This impairment can be particularly consequential in safety-sensitive professions, where attention and decision-making are crucial for minimising risks and preventing accidents.

Beyond alcohol, several commonly prescribed medications have been shown to markedly impair cognitive performance in a similar dose-dependent manner, most notably benzodiazepines (Huizinga et al., 2019). Benzodiazepines, which are predominantly used therapeutically for anxiolytic and hypnotic purposes (i.e. for anxiety and insomnia, respectively), rank among the most widely prescribed medications (e.g. alprazolam, diazepam, temazepam; Australian Institute of Health and Welfare, 2022; Bachhuber et al., 2016; Santo et al., 2020). While benzodiazepines have clear benefits in the context of surgical premedication or for those with medical conditions, it is important to acknowledge that their use can lead to impairment in a variety of cognitive processes (Dassanayake et al., 2011). For instance, a 1 mg dose of alprazolam has been shown to slow reaction time, compromise motor coordination, and diminish attentional capacities (Crowe and Stranks, 2018; Huizinga et al., 2019). It is crucial to acknowledge that these medications may considerably hinder the daily functioning of individuals consuming them recreationally or on an outpatient basis, especially in naïve users. Despite the known cognitive impacts of both drugs and the high prevalence of co-consumption (Ilomäki et al., 2013), most research has considered the impact of alcohol and benzodiazepines in isolation.

Studies that have investigated their combined use offer inconsistent findings, with some studies suggesting an additive (Roehrs, 2001; van Steveninck et al., 1993) or possibly synergistic effect (Kunsman et al., 1992; Linnoila and Mattila, 1973; Mørland et al., 1974) when used in combination while others have found no such interaction (Bond et al., 1992; Landauer et al., 1974; Linnoila et al., 1990; Seppälä et al., 1982; Taeuber et al., 1979). Given that as many as 88% of benzodiazepine users report the additional consumption of alcohol, and 28% indicate intentional combined use (Ilomäki et al., 2013), there is an urgent need for a more comprehensive understanding of the combined effects on cognitive functioning. This study aimed to build upon existing research by employing a robust within-subject, randomised, placebo-controlled design to directly compare the acute effects of a moderate dose of alcohol and 1 mg of alprazolam, alone and in combination, on cognitive performance. Addressing this gap is essential for informing clinical practise, public health policies and preventative interventions.

## Methods

### Participants

Healthy volunteers aged 21–40 years were recruited in Melbourne, Australia through physical flyers and online advertisements. Inclusion criteria included normal or corrected-to-normal vision, a body mass index (BMI) between 18.5 and 29.9 kg/m^2^, and a blood pressure lower than 160/100 mmHg. Individuals with a current or past diagnosis of physical, gastrointestinal, neurological or psychiatric condition were excluded, as were those who reported current use of any medication (unless approved by the study doctor), high-risk alcohol consumption (as determined by the alcohol use identification test; Barbor et al., 2001) or were pregnant. Participants were asked to refrain from consuming food or drinks (except water) within 2 h of testing, caffeine-containing products within 12 h of testing, alcohol and nicotine within 24 h of testing or psychoactive drugs or medications (unless approved by investigators) within 7 days of testing. All participants provided written consent before participating in the study and were reimbursed for their time.

### Study design and procedures

This study was part of a larger investigation consisting of three separate randomised, double-blind, placebo-controlled, crossover trials aimed at examining the effects of alcohol (0.95 g/kg) and three commonly prescribed benzodiazepines (1 mg alprazolam, 1 mg temazepam and 5 mg diazepam) on driving performance (Australian New Zealand Clinical Trials Registry (ANZCTR, http://www.anzctr.org.au) number ACTRN12618000727246). Within the framework of this larger investigation, the present study presents an analysis of secondary endpoints, focusing on the combined effect of alcohol and alprazolam on cognitive function.

This study involved five visits to Swinburne University’s Centre for Human Psychopharmacology, with the first visit for screening and familiarisation and the following four as experimental sessions spaced at least 7 days apart. Prior to enrolment, candidates were assessed for eligibility through medical history and physical examination, and practised the cognitive assessments. On testing days, participants arrived between 9:00 a.m. and 12:00 p.m., reconfirmed their eligibility, underwent a baseline breathalyser to confirm a zero BAC and were screened for tetrahydrocannabinol, benzodiazepines, cocaine, amphetamines and opiates using a Securetec 6S DrugWipe. Participants were administered each of the following four treatments across different sessions: (1) placebo beverage plus placebo capsule; (2) 0.95 g/kg alcohol plus placebo capsule; (3) placebo beverage plus 1 mg alprazolam or (4) 0.95 g/kg alcohol plus 1 mg of alprazolam. Treatments were randomly assigned by a disinterested third party (research coordinator), and both investigators and participants were blind to the assignment. After a 30-min absorption period, participants provided BAC readings and blood samples, reported subjective drug effects using the brief biphasic alcohol effects scale (B-BAES; Rueger and King, 2013) and completed the cognitive assessment. Following the cognitive assessment, participants provided another breathalyser reading and again reported subjective drug effects. At 100 min post-dosing, participants completed an identical regime before being sent home via taxi. Testing visits occurred at the same time each week to control for circadian variation in performance. The study protocol was approved by the Swinburne University Human Research Ethics Committee in compliance with the National Statement on Ethical Conduct in Human Research (2018).

### Study treatments

Identical capsules containing either 1 mg of alprazolam or placebo were provided by a compounding pharmacy (MyCompounder, Australia). The alcohol treatment consisted of Absolut^TM^ vodka (0.95 g/kg of body weight) mixed with 300 mL of orange juice. The placebo beverage consisted of orange juice only, with alcohol wiped around the rim of the cup to aid in the double-blind nature of this study (Benson et al., 2019; Benson and Scholey, 2014; Garrisson et al., 2022). A trial nurse prepared both treatments away from the investigator and participant.

### Alcohol and alprazolam analysis

BAC was measured at baseline, and again at 30, 50, 100 and 120 min post-dosing using a Lion Alcolmeter SD-400PA breathalyser (Lion Laboratories Limited, 2018). Calibrated quarterly at the road policing drug and alcohol section of Victoria police, this breathalyser measures breath alcohol concentration, which is converted to BAC, calculated at a ratio of 2300:1.

Venous whole-blood samples (10 mL) were collected at 30 and 100 min post-dosing using a BD Vacutainer^®^ tube containing 100 mg sodium fluoride and 20 mg potassium oxalate from the cubital fossa region on the non-dominant arm via single-draw venepuncture. All blood samples were immediately stored at −80°C until collection by a courier for shipment to Forensic Science SA, Australia, for analysis. Blood samples were initially analysed singly, with positive samples subsequently analysed in duplicate. Calibration standards were prepared that ranged from 1 to 32 ng/mL for alprazolam (Supelco), using D5-alprazolam (Cerilliant) as the internal standard spiked at a concentration of 25 ng/mL. Samples (500 µL) were diluted with ammonium hydroxide (0.1% in water, 500 µL, Chem Supply), ethanol (160 µL, Ajax Finechem) and internal standard (25 µL, 500 ng/mL) and vortexed. An aliquot (800 µL) was added to the SLE cartridge (Biotage ISOLUTE SLE 1 mL supported liquid extraction column) and after 5 min absorption, the samples were eluted with isopropanol:dichloromethane (5:95, 2.5 mL, Chem Supply) and allowed to flow under gravity for 5 min. Positive pressure was applied to elute any remaining extraction solvent. Methyl tert-butyl ether (2.5 mL, Sigma Aldrich) was then added and allowed to flow under gravity for 5 min. Positive pressure was applied to elute any remaining extraction solvent. The combined eluants were evaporated to dryness and reconstituted in ethanol (40 µL, Ajax Finechem) and formic acid (0.1% in water, 40 µL, Optima) and transferred to limited volume LC vials. These were capped and centrifuged (3000 rpm, 5 min), and analysed on an Agilent 6546 quadrupole time-of-flight mass spectrometer connected to an Agilent 1290 infinity II liquid chromatography system fitted with a waters acquity ethylene bridged hybrid C18 (1.7 µM, 3.0 mm × 50 mm) column. The mobile phase was a gradient of acetonitrile (Optima LCMS grade) and 0.1% formic acid (Optima) over 12 min with a flow rate of 0.35 mL/min. Calibration was linear (*r*^2^ > 0.99) with a 1/x weighting applied without forcing through zero. The lower and upper limits of quantitation were 1 and 32 ng/mL, respectively. The limit of detection was 0.5 ng/mL.

### Cognitive assessment

Cognitive function across domains of attention, memory and response speed was assessed using the CogTrack (Wesnes et al., 2017) and CogPro (Ecog Pro Ltd., Bristol, UK) online computerised test batteries at 30 and 100 min after drug administration. The following nine tasks took approximately 20 min to complete and were presented in a standardised form on a study computer in a quiet environment.

#### Simple reaction time

Participants quickly respond to the word ‘yes’ on the screen by pressing the right arrow key with their right forefinger. The word appears randomly, with 50 stimuli presented at intervals of 1–3.5 s. The task measures response speed (ms) and takes about 2 min.

#### Choice reaction time

Participants place their forefingers on the arrow keys and respond to 50 unpredictable stimuli in the centre of the screen. They must press the correct key as quickly and accurately as possible. The stimuli have random intervals of one to three and a half seconds. The task measures response speed (ms) and accuracy (%), taking approximately 2 min.

#### Digit vigilance

Participants monitor a stream of digits presented on the screen and press the right arrow key whenever a target digit appears, regardless of visibility. The task measures the speed (ms) of correct detections, accuracy (%) and the number of false alarms. Digits are presented at a rate of 150/min for 3 min.

#### Spatial working memory

Participants memorise a three-by-three lit bulb pattern for 10 s and then respond to 36 random ‘probe’ stimuli. They press the right arrow key for the originally lit bulbs and the left arrow key for non-target positions. The task measures speed (ms) and accuracy (%), taking about one and a half minutes.

#### Numeric working memory

Participants are shown a series of five digits and must recall them when presented with 30 probe digits. They press the right arrow key for target digits and the left arrow key for non-target digits. The task measures speed (ms) and accuracy (%), taking approximately 1.5 min.

#### Word recall and recognition

Participants are shown 15 words and must recall as many as possible within 60 s. They also engage in delayed word recall and recognition, typing words they remember within 60 s and distinguishing between original words and distractor words. Speed (ms), accuracy (%) and number of errors are measured. The task takes around 4 min in total.

#### Picture recognition

Participants view 20 pictures on a screen, with one picture displayed every 3 s. After 15 min, the original pictures are shown again, mixed with 20 lure pictures. Participants press the right arrow key for originals and the left arrow key for lures. The task measures speed (ms) and accuracy (%), using different picture pairs at each visit. It takes approximately 3.5 min.

For reliability and normative values, please refer to Wesnes et al. (2017). To manage practise effects, all participants were required to complete the cognitive assessment twice during an acclimatising session that occurred prior to the scheduled testing days. The training data were recorded but not analysed.

### Brief biphasic alcohol effects scale

The B-BAES questionnaire was used to assess subjective drug effects before and after each cognitive assessment at 30, 50, 100 and 120 min post-dosing. The B-BAES is divided into two subscales, sedative and stimulative, each with three items. On an 11-point scale, participants rated these items from 0 (not at all) to 10 (extremely). The scores for each subscale were determined by summing the ratings of the three respective items. The B-BAES is a validated measure that has been used in several studies to assess the sedative and stimulant effects of various drugs, such as cannabidiol (Spinella et al., 2021), caffeinated energy drinks (Cheng et al., 2017) and nitrous oxide (Kamboj et al., 2021).

### Statistical analysis

Descriptive analysis was used to assess participant demographics and substance use history. Linear fixed-effects models with restricted maximum likelihood were employed to examine the impact the of condition (placebo, alcohol, alprazolam and combined alcohol and alprazolam), time (30 and 100 min) and their interaction on cognitive performance and subjective mood. Compound symmetry was selected as the optimal variance structure based on Akaike’s information criterion. Initially, condition by time interactions were examined. In the absence of a significant interaction, an exploratory analysis of the main effects was conducted, and Bonferroni-corrected pairwise comparisons were utilised to compare treatment effects with the placebo condition. Outliers were screened in the cognitive data prior to analysis. An exploratory approach was taken for outlier handling, considering factors such as reaction time patterns and performance consistency. Values influenced by factors external to the intended experimental conditions, such as distraction or equipment malfunction, were removed from the dataset. A sensitivity analysis was performed to assess the impact of outliers on the findings, which showed minimal or slightly more significant differences when including outliers. Statistical analyses were conducted using IBM SPSS Statistics (Version 29), and all tests were two-tailed with a significance level of *p* < 0.05.

## Results

**Figure 1.**
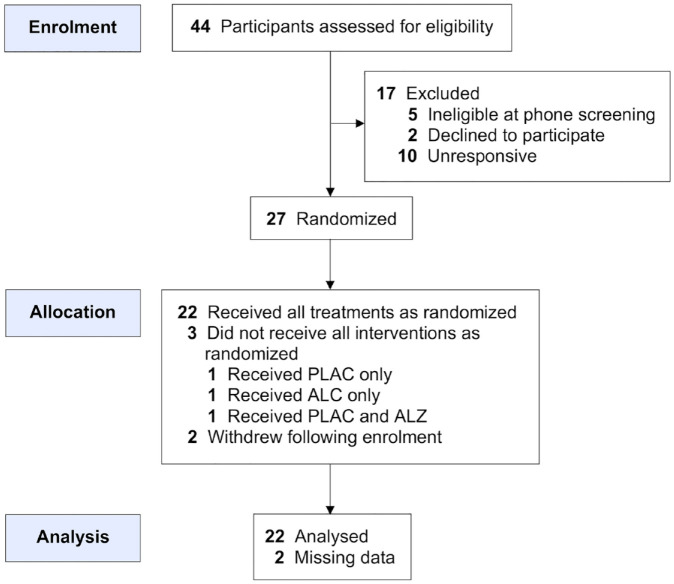
Adapted CONSORT diagram illustrating the flow of participants through the study.

Of 44 individuals assessed for eligibility, five were found to be ineligible, two declined to participate and 10 did not respond to the invitation. The remaining 27 participants were enrolled and randomised into the study between August 2018 and April 2022. Two participants subsequently withdrew following enrolment without consuming any treatment, and three did not receive all four treatments (Figure 1). Upon data analysis, two participants were found to have substantial missing data, leading to their exclusion from the dataset. The final sample comprised 12 females and eight males, aged between 21 and 38 years (mean age = 28.6 ± 4.0 years) and a mean BMI of 24.2 ± 3.3. Participant demographics and substance use history are detailed in Table 1.

**Table 1.** Demographic characteristics and drug-use history (*N* = 20).

Demographics	*n* (%)
Sex	
Female	12 (60%)
Male	8 (40%)
Age group (years)	
Less than 25	1 (5%)
25–35	16 (80%)
35–40	3 (15%)
Education status^a^	
Secondary	2 (10%)
Tertiary	13 (65%)
Postgraduate	5 (25%)
Employment status	
Student	4 (20%)
Part time/casual	7 (35%)
Full time	6 (30%)
Unemployed	3 (15%)
Drug-use history^b^	
Alcohol	20 (100%)
3,4-Methylenedioxymethamphetamine	13 (65%)
Cannabis	19 (95%)
Amphetamines	12 (60%)
Cocaine	10 (50%)
Heroin	–
Frequency of alcohol use	
Daily or almost daily	6 (30%)
1–2 times a week	5 (25%)
2–3 times a month	4 (20%)
Monthly or less	5 (25%)
Typical quantity consumed (*n* (%))^c^	
1–4	13 (65%)
5–9	4 (20%)
10 or more	1 (5%)

a Highest education level completed.

b Positive indication of past use.

c Typical number of drinks consumed on drinking days.

### Blood concentration of alcohol and alprazolam

Table 2 presents the average BAC and whole-blood alprazolam concentrations at various sampling time points. Peak BAC was 0.030% following the alcohol treatment and 0.031% when alcohol was taken in combination with alprazolam, both at 30 min after drug administration. Both alcohol treatments (alone and when taken in combination with alprazolam) result in similar BACs across time points (all *p* > 0.05), decreasing from 50 to 100 min (both *p* < 0.001) and again from 100 to 120 min post-dosing (*p* < 0.001 and 0.003, respectively). Whole-blood alprazolam concentration increased significantly from 30 to 100 min when alprazolam was taken alone and in combination with alcohol (both *p* < 0.001). Although whole-blood concentration was slightly higher when alprazolam was taken with alcohol compared to being consumed alone at both time points, these differences were not significant (*p* *>* 0.05).

**Table 2.** Mean (±SD) BACs (%) and whole-blood alprazolam concentrations (mg/L) across condition and sampling time points (*N* = 20).

Time (min)	BAC (%)		Whole-blood alprazolam concentration (mg/L)*	
	Alcohol	Alcohol and alprazolam	Alprazolam	Alcohol and alprazolam
30	0.030 (0.011)	0.031 (0.014)	1.74 (4.08)	2.76 (4.79)
50	0.027 (0.010)	0.028 (0.015)	–	–
100	0.016 (0.011)	0.009 (0.013)	8.79 (3.10)	10.06 (3.45)
120	0.006 (0.010)	0.004 (0.009)	–	–

BAC: blood alcohol concentration; Time: time since drug administration.

* *N* = 18.

### Cognitive performance

Table 3 presents summary data, including means and standard deviations (±), for cognitive performance outcomes. For brevity, only significant interactions, main effects and pairwise comparisons are reported.

**Table 3.** Mean (±SD) cognitive performance scores across condition and time (*N* = 20).

Outcome	Placebo			Alcohol			Alprazolam			Alcohol and alprazolam		
	Mean (SD)			Mean (SD)			Mean (SD)			Mean (SD)		
	30 min	100 min	Overall	30 min	100 min	Overall	30 min	100 min	Overall	30 min	100 min	Overall
Reaction time												
Simple reaction time (ms)	292.11 (33.60)	294.79 (39.96)	293.48 (36.54)	303.13 (47.87)	296.87 (35.96)	300.08 (42.05)	295.99 (35.87)	315.82 (54.77)^a^	305.65 (46.53)	297.40 (34.18)	320.28 (50.53)^a^*	308.84 (44.13)
Choice reaction time (ms)	426.20 (56.68)	431.07 (56.53)	428.63 (55.93)	437.52 (78.96)	428.32 (68.63)	432.92 (73.17)	444.33 (78.07)	462.51 (84.58)	452.94 (80.63)^a^	446.87 (75.26)	472.68 (102.09)	459.77 (89.49)^a^*,^b^
Choice reaction time accuracy (%)	95.20 (3.00)	97.10 (2.55)	96.15 (2.91)	94.30 (4.17)	96.32 (3.61)	95.28 (3.99)	94.40 (4.13)	92.89 (4.24)^a^*,^b^	93.68 (4.20)	94.00 (4.15)	92.80 (4.87)^a^*,^b^*	93.40 (4.51)
Digit vigilance												
Reaction time (ms)	454.89 (54.23)	456.58 (53.00)	455.73 (52.93)	465.30 (60.02)	466.86 (59.21)	466.08 (58.85)	465.33 (50.57)	485.37 (64.40)	475.35 (58.05)^a^	480.31 (48.43)	500.57 (51.43)	490.44 (50.37)^a^*
Accuracy (%)	95.87 (4.49)	96.53 (5.50)	96.20 (4.97)	97.21 (3.55)	95.12 (5.80)	96.16 (4.86)	93.08 (6.57)	90.66 (7.57)	91.94 (7.07)^a^*	95.43 (5.32)	90.52 (6.53)	92.98 (6.39)^a^
False alarms (#)	0.55 (1.05)	0.75 (1.48)	0.65 (1.27)	0.35 (0.67)	0.50 (0.83)	0.42 (0.75)	0.63 (1.30)	1.11 (1.94)	0.87 (1.65)	0.55 (0.89)	1.24 (1.92)	0.86 (1.48)
Numeric working memory												
Reaction time (ms)	664.66 (125.53)	632.92 (100.28)	648.79 (113.29)	627.89 (115.05)	607.72 (142.53)	617.81 (128.26)	704.40 (145.76)^a^*	749.42 (180.39)^a^*,^b^*	727.49 (158.43)	651.01 (131.01)	749.08 (211.62)^a^*,^b^*	701/30 (181.63)
Accuracy (%)	94.39 (4.65)	96.44 (2.66)	95.42 (3.88)	95.72 (3.65)	93.72 (4.04)	94.72 (3.93)	93.68 (5.08)	93.10 (5.25)	93.39 (5.10)	91.50 (7.63)	91.40 (6.60)	91.45 (7.06) ^a^*,^b^
Spatial working memory												
Reaction time (ms)	655.13 (203.45)	577.03 (119.97)	617.08 (170.52)	605.15 (139.24)	563.27 (93.26)	584.21 (118.88)	692.19 (204.93)	667.57 (188.23)	680.19 (194.77)^a,b^*	631.24 (170.64)	644.83 (182.63)	637.68 (174.62)^b^
Accuracy (%)	96.63 (2.66)	96.09 (4.28)	96.36 (3.51)	93.94 (5.72)	93.97 (6.07)	93.95 (5.82)	90.75 (9.34)	90.94 (6.05)	90.84 (7.77)^a^*	94.18 (6.43)	91.49 (6.54)	92.87 (6.54)^b^
Immediate word recall												
Accuracy (%)	55.33 (21.86)	50.00 (21.47)	62.67 (21.55)	49.00 (19.38)	56.67 (20.58)	52.83 (20.11)	50.67 (20.10)	39.67 (21.57)^b^*	45.17 (21.32)	48.00 (23.55)	41.00 (23.42)^b^*	44.50 (23.46)
Errors (#)	0.25 (0.55)	0.70 (0.87)	0.47 (0.75)	0.70 (0.86)	0.40 (0.68)	0.55 (0.78)	0.45 (0.76)	0.42 (0.69)	0.44 (0.72)	0.80 (0.95)	0.79 (0.98)	0.79 (0.95)
Delayed word recall												
Accuracy (%)	48.00 (24.62)	35.67 30.46)	41.83 (28.04)	38.33 (21.51)	44.00 (26.79)	41.17 (24.15)	39.33 (25.68)	18.00 (24.19)^a^*	28.67 (26.89)	36.67 (27.06)	21.67 (30.88)^a,b^*	29.17 (29.65)
Errors (#)	0.90 (1.37)	1.10 (1.25)	1.00 (1.30)	1.16 (1.42)	0.80 (1.11)	0.97 (1.27)	0.70 (1.03)	1.90 (1.77)^b^*	1.30 (1.56)	0.85 (1.04)	1.37 (1.42)	1.10 (1.25)
Word recognition												
Reaction time (ms)	806.93 (186.39)	814.13 (212.83)	810.53 (197.50)	812.79 (221.40)	756.57 (160.43)	789.79 (193.06)	875.25 (225.80)	857.12 (208.82)	866.67 (215.18)^a^*	784.87 (175.81)	838.94 (184.25)	811.90 (179.85)
Accuracy (%)	82.83 (12.20)	83.17 (10.34)	83.00 (11.17)	80.83 (14.82)	83.67 (13.59)	82.25 (14.11)	79.83 (13.53)	75.26 (16.86)	77.61 (15.22)^a^	80.50 (15.00)	75.67 (17.97)	78.08 (16.52)^a^
Picture recognition												
Reaction time (ms)	1027.84 (255.83)	982.78 (234.48)	1005.31 (243.30)	979.42 (205.59)	936.49 (219.31)	957.96 (210.43)	1015.95 (184.45)	1049.72 (200.76)	1032.83 (191.06)	1021.77 (249.88)	1022.96 (243.31)	1002.35 (243.44)
Accuracy (%)	83.25 (11.98)	80.63 (11.83)	81.94 (11.83)	82.38 (9.95)	82.75 (9.63)	82.56 (9.67)	84.13 (8.93)	71.25 (14.81)^a, b^*	77.69 (13.72)	80.88 (11.73)	78.42 (14.20)	79.68 (12.88)

All pairwise comparisons were Bonferroni corrected for multiple comparisons.

Ms: milliseconds; #: number.

a Significant difference compared to placebo at *p* < 0.05; with *p < 0.01.

b Significant difference compared to alcohol condition at *p* < 0.05; with *p < 0.01.

#### Simple reaction time

There was a significant condition by time interaction for simple reaction time (ms) (*F*_(3,129.78)_ = 4.09, *p* = 0.020), with a main effect of the condition occurring at 100 min (*F*_(3,54.71)_ = 5.65, *p* = 0.002).

**Figure 2.**
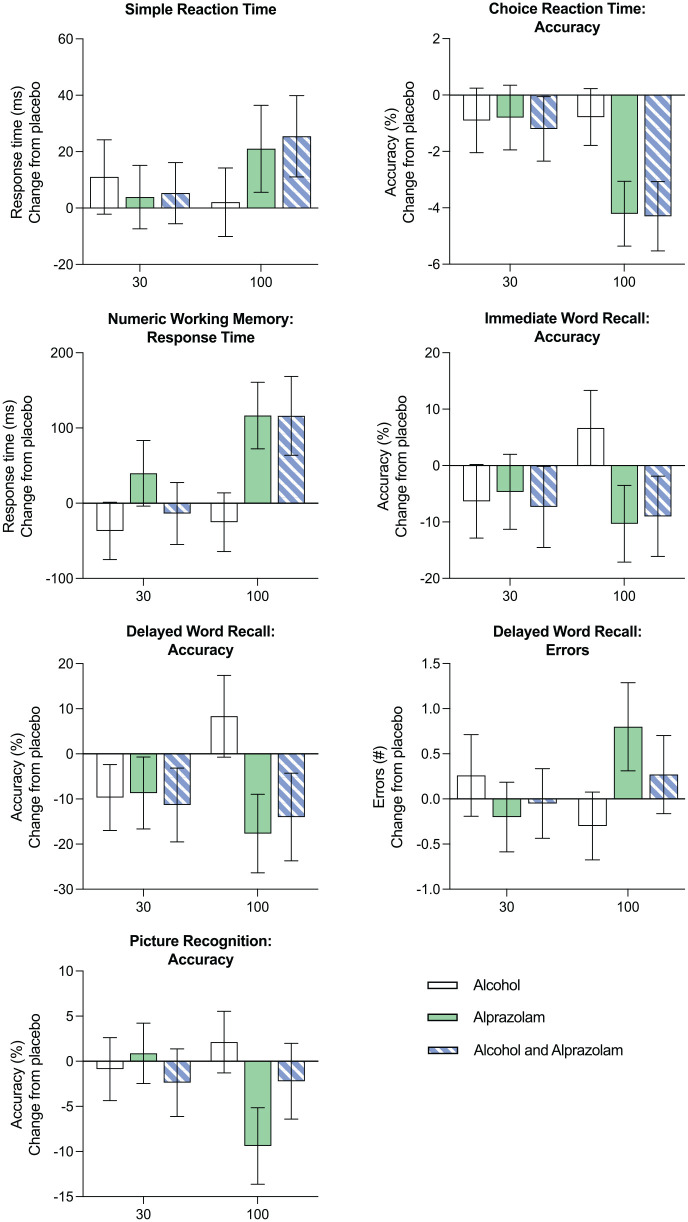
Mean change from placebo across conditions (alcohol, alprazolam and combined alcohol and alprazolam) and time (30 and 100 min) for simple reaction time (ms), choice reaction time (ms), numeric working memory reaction time (ms), immediate word recall accuracy (%), delayed word recall accuracy (%) and picture recognition accuracy (%). Errors bars indicate ± standard error of the mean (SEM).

At 100 min, reaction time (ms) increased (poorer performance) in the alprazolam (*p* = 0.012) and the alcohol and alprazolam conditions relative to placebo (*p* = 0.009) (Figure 2).

#### Choice reaction time

Condition effects were evident for choice reaction time (*F*_(3,131.04)_ = 5.86, *p* < 0.001) with significantly poorer performance (increased reaction time) in the alprazolam (*p* = 0.025) and alcohol and alprazolam conditions compared to placebo (*p* = 0.005). Performance also decreased in the alcohol and alprazolam condition relative to the alcohol condition (*p* = 0.024). Moreover, a significant condition by time interaction was found for choice reaction time accuracy (*F*_(3,130.12)_ = 3.70, *p* = 0.014), with a main effect of condition at 100 min (*F*_(3,54.56)_ = 10.34, *p* < 0.001). At 100 min, performance decreased (fewer correct responses) in the alprazolam and the alcohol and alprazolam conditions relative to both the placebo (both *p* < 0.001) and alcohol (*p* = 0.013 and 0.004, respectively) conditions (Figure 2).

#### Digit vigilance

There was a main effect of condition for digit vigilance reaction time (*F*_(3,133)_ = 10.26, *p* < 0.001). Relative to placebo, reaction time increased (poorer performance) in the alprazolam (*p* = 0.018) and the alcohol and alprazolam condition (*p* < 0.001). There was also a main effect of condition for digit vigilance accuracy (*F*_(3,130.96)_ = 8.20, *p* < 0.001), with poorer performance (fewer correct responses) in the alprazolam (*p* < 0.001) and the alcohol and alprazolam condition compared to placebo (*p* = 0.027). In addition, compared to the alcohol condition, accuracy declined (poorer performance) in the alprazolam (*p* < 0.001) and the alcohol and alprazolam condition (*p* = 0.030). There was a main effect of time across all measures of digit vigilance, with reaction time and false alarms increasing, and accuracy decreasing from 30 to 100 min (*p* = 0.019, 0.001 and 0.005, respectively).

#### Numeric working memory

A significant condition by time interaction was found for numeric working memory reaction time (*F*_(3,130.92)_ = 3.46, *p* = 0.018), with main effects of condition at 30 min (*F*_(3,54.82)_ = 3.31, *p* = 0.027) and 100 min (*F*_(3,57)_ = 11.25, *p* < 0.001). At 30 min, reaction time increased (reduced performance) in the alprazolam condition relative to placebo (*p* = 0.016). At 100 min, performance declined (increased reaction time) in the alprazolam and the alcohol and alprazolam conditions compared to the placebo (both *p* = 0.003) and alcohol conditions (both *p* < 0.001) (Figure 2). In addition, there was a main effect of the condition on numeric working memory accuracy (*F*_(3,130.13)_ = 5.92, *p* < 0.001), with poorer performance (reduced accuracy) in the alcohol and alprazolam condition relative to both placebo (*p* < 0.001) and alcohol conditions (*p* = 0.010).

#### Spatial working memory

Condition effects were evident for spatial working memory reaction time, with slower reaction time (poorer performance) in the alprazolam condition compared to the placebo (*p* = 0.040) and alcohol conditions (*p* < 0.001). Reaction time also increased in the alcohol and alprazolam condition compared to the alcohol condition (*p* = 0.044). Moreover, there was a main effect of the condition for spatial working memory accuracy (*F*_(3,129.351)_ = 7.85, *p* < 0.001), with fewer correct responses (reduced accuracy) made in the alprazolam condition compared to placebo (*p* < 0.001). Accuracy also decreased in the alcohol and alprazolam condition relative to the alcohol condition (*p* = 0.049).

#### Immediate word recall

There was a significant condition by time interaction for immediate word recall accuracy (*F*_(3,133)_ = 4.21, *p* = 0.007), with a main effect of condition at 100 min (*F*_(3,57)_ = 8.47, *p* < 0.001). At 100 min, performance declined (accuracy declined) in the alprazolam (*p* < 0.001) and the alcohol and alprazolam condition relative to the alcohol condition (*p* = 0.001) (Figure 2).

#### Delayed word recall

A significant condition by time interaction was found for delayed word recall accuracy (*F*_(3,133)_ = 5.50, *p* = 0.001). An effect of condition on accuracy was found at 100 min (*F*_(3,57)_ = 12.74, *p* < 0.001), whereby performance declined (fewer correct responses) in the alprazolam and the alcohol and alprazolam condition compared to both placebo (*p* = 0.003 and 0.031, respectively) and alcohol conditions (both *p* < 0.001). There was also a significant condition by time interaction for delayed word recall errors (*F*_(3,131.10)_ = 4.15, *p* = 0.008), with a main effect occurring at 100 min (*F*_(3,56.14)_ = 4.04, *p* = 0.011). At 100 min, performance declined (more incorrect responses) in the alprazolam condition relative to the alcohol condition (*p* = 0.009) (Figure 2).

#### Word recognition

There was a significant main effect of the condition on word recognition reaction time (*F*_(3,129.97)_ = 3.97, *p* = 0.010), with greater reaction times (poorer performance) in the alprazolam condition compared to placebo (*p* = 0.008). Moreover, a significant main effect of condition was observed for accuracy (*F*_(3,132.03)_ = 4.51, *p* = 0.005) with reduced performance (fewer correct responses) in the alprazolam (*p* = 0.027) and the alcohol and alprazolam condition relative to placebo (*p* = 0.049).

#### Picture recognition

A significant condition by time interaction was found for picture recognition accuracy (*F*_(3,131.99)_ = 4.50, *p* = 0.005), with a main effect of condition at 100 min (*F*_(3,56.08)_ = 4.64, *p* = 0.006). At 100 min, accuracy declined (poorer performance) in the alprazolam condition relative to both placebo (*p* = 0.036) and alcohol conditions (*p* = 0.005) (Figure 2).

### Brief biphasic alcohol effects scale

**Figure 3.**
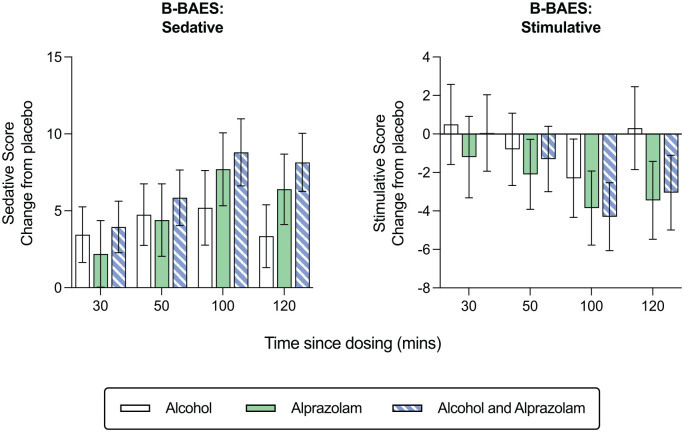
Mean change from placebo across condition (alcohol, alprazolam and combined alcohol and alprazolam) and time (30, 50, 100 and 120 min) for sedative and stimulative B-BAES subscale scores. Errors bars indicate ± standard error of the mean (SEM).

Figure 3 shows the mean (±SD) scores for sedative and stimulative subscales of the B-BAES across time points for the four conditions. There was a significant main effect of condition on the sedative score (*F*_(3,285)_ = 31.27, *p* < 0.001), with sedation increasing in the alcohol, alprazolam, and the alcohol and alprazolam condition relative to placebo (all *p* < 0.001). Similarly, there was a main effect of the condition on the stimulative score (*F*_(3,285)_ = 9.24, *p* < 0.001), with stimulation decreasing in the alcohol (*p* < 0.001) and the alcohol and alprazolam condition compared to placebo (*p* = 0.002). Moreover, there was a main effect of time on sedative (*F*_(3,285)_ = 12.07, *p* < 0.001) and stimulative scores (*F*_(3,285)_ = 31.99, *p* < 0.001). Sedation increased and stimulation decreased from 30 to 60 min after drug administration (*p* = 0.005 and *p* < 0.001, respectively). Stimulation also decreased from 60 to 100 min post-dosing (*p* < 0.001).

## Discussion

Our research findings indicate that a low dose of alcohol (0.03% BAC) does not lead to cognitive impairment across a range of tasks. These results are consistent with previous studies that have shown limited cognitive effects at lower alcohol levels when consumed in isolation (Garrisson et al., 2021). For instance, deficits in reaction time typically become apparent at higher BACs (e.g. 0.08–0.106%; Hindmarch and Gudgeon, 1982; Liguori et al., 1999) but are less likely to be observed within the range of 0.03–0.05% (Breitmeier et al., 2007). In addition, while some research has reported impairments in executive function at a 0.08% BAC (Charlton and Starkey, 2015; Starkey and Charlton, 2014), others have found no such deficits between 0.04% and 0.065% (Hoffman and Nixon, 2015). Similarly, sustained attention deficits have been detected at BACs of 0.08% and 0.103%, but not between 0.024% and 0.057% (Hindmarch and Gudgeon, 1982).

There were consistent negative effects on cognitive performance following the administration of 1 mg of alprazolam, aligning with previous research (Huizinga et al., 2019; Leufkens et al., 2007; Scavone et al., 1992; Vermeeren et al., 1995). These effects were most pronounced approximately 100 min after dosing, coinciding with the expected peak concentration of alprazolam. Interestingly, although the combination of alprazolam and alcohol resulted in higher blood concentrations of alprazolam, we did not observe any discernible differences in cognitive performance compared to alprazolam alone. This study contributes valuable data to the limited body of research that examines the effects of commonly consumed therapeutic doses of alprazolam in healthy individuals. While our investigation focused on a 1 mg dose of alprazolam, it is noteworthy that similar impairments have been observed in studies using different doses. For example, early research by Rush and Griffiths (1997) reported impairments in digit entry and recall tasks following 1.5 mg of alprazolam, and Suzuki et al. (1995) found that a 0.8 mg dose negatively affected vigilance performance 3 h after consumption. Furthermore, Allen et al. (1991) observed impaired word recall with doses ranging from 0.25 to 0.75 mg at 3 h post-dosing, with effects persisting for up to 10 days of cumulative treatment.

Alcohol, alprazolam and their combination resulted in significant but not additive increases in self-reported sedation compared to placebo. In addition, compared to placebo, stimulation decreased with alprazolam alone and when taken in combination with alcohol. Although a moderate dose of alcohol typically induces stimulation on the rising BAC limb (Holland and Ferner, 2017), this response was absent in our study. The absence of stimulation may be due to the lower alcohol dose used (i.e. 0.03% BAC) or may reflect the timing of the scales and the inability to capture this effect under the methodology used. Lower doses of alcohol may not reach the threshold necessary to activate neurotransmitter systems, such as dopamine and serotonin, both of which play crucial roles in creating stimulative effects (Cheng et al., 2017). Nevertheless, the sensitivity of the B-BAES to alprazolam intoxication highlights the perceived similarities in the intoxicating effects of these two drug classes. When evaluating the absence of an alprazolam–alcohol interaction on measures of performance and mood, it is important to consider the potential influence of pharmacokinetic factors. One possible explanation for the absence of synergistic or additive effects is the misalignment in the timing of peak concentrations for each substance. Alprazolam typically reaches its highest concentration within 1–2 h after administration (Moody, 2012), while the peak alcohol occurred earlier, as indicated in Table 2. The non-overlapping peak concentrations of alprazolam and alcohol may have precluded their respective peak effects from coinciding, explaining the lack of an interactive effect on cognitive performance observed in our study. Individuals who intentionally or unintentionally time their consumption to align the peaks of the alprazolam and alcohol, however, may experience exacerbated impairments to cognitive and psychomotor abilities. The heightened impairment could significantly increase the risk of difficulties in various tasks, such as driving, thus compromising safety.

The current study presents some limitations. Firstly, our findings may have limited generalisability to clinical populations as we focused on investigating the effects of alcohol and alprazolam on cognitive performance in a non-clinical sample. Clinical populations, who are more likely to be prescribed alprazolam, may exhibit different effects due to underlying mental health conditions or variations in drug metabolism. Secondly, our study specifically focused on the immediate effects of alcohol and alprazolam without considering the long-term or cumulative effects of their combined usage on cognitive function. Research has shown that long-term users of benzodiazepines may experience cognitive impairment even after discontinuation of the medication (Crowe and Stranks, 2018; van der Sluiszen et al., 2019). This suggests that the co-consumption of alcohol and benzodiazepines remains risky regardless of long-term use, due to the potential for drug–drug interactions that may not be readily anticipated by individuals engaging in such behaviour. In addition, it is worth noting that all of our individual tests were of short duration, lasting between one and a half to 4 min, which might have reduced sensitivity to the impact of alcohol. By contrast, more extended and complex cognitive assessments may better identify subtle alcohol-induced impairments or declines in vigilance that our chosen tasks did not capture.

A 1 mg therapeutic dose of alprazolam, whether taken alone or in combination with alcohol, significantly impaired cognitive function, with the most pronounced effects observed 100 min post-consumption. While laboratory tests may not directly reflect specific real-world task performance, they provide valuable insights into the effects of drugs on various cognitive and behavioural components involved in everyday tasks. It is important to assess different aspects of cognitive performance to identify critical cognitive processes vulnerable to impairment by commonly consumed substances. Future research should investigate varying doses and administration timings of alcohol and benzodiazepines on cognitive performance, as well as explore the potential impact of chronic alcohol and benzodiazepine use, including tolerance and sensitisation effects. Although we did not identify an interactive or additive effect of alcohol and alprazolam, our findings that a therapeutic or moderate dose of alprazolam impaired all aspects of cognitive performance holds valuable implications, especially considering the prevalence of non-medical use (Votaw et al., 2019). This highlights the potential risks associated with the widespread use of alprazolam and the need for further research and awareness regarding its cognitive effects. These findings also have significant implications for public health and the development of policies and guidelines regarding the use of prescription medication and alcohol, considering their potential impact on cognitive impairment in daily activities.
